# A Case Report of Low-Dose Steroid Induced Psychosis in an Older Adult with Rheumatoid Arthritis

**DOI:** 10.1192/j.eurpsy.2023.537

**Published:** 2023-07-19

**Authors:** N. Edokobi, S. Sreevalsam Anil, J. Wells

**Affiliations:** ^1^ Virginia Tech Carilion School of Medicine; ^2^Department of Psychiatry, Carilion Roanoke Memorial Hospital/Virginia Tech Carilion School of Medicine, Roanoke, United States

## Abstract

**Introduction:**

Rheumatoid arthritis (RA) is typically known for its intra-articular manifestations in the joints, and steroids are considered one of the first-line medications for it. Steroids are known for neuropsychiatric manifestations, but it is rarely reported in low-dose steroids.

**Objectives:**

We describe a case of psychosis in an older adult with RA precipitated by low-dose prednisone with no past history of neuropsychiatric symptoms from steroids in the past five years.

**Methods:**

Miss X is a 63-year-old female with past history of RA, major depressive disorder, hypothyroidism, chronic obstructive pulmonary disease, and hypertension, presented with one-week history of irritable mood, increased psychomotor agitation, decreased need for sleep and appetite, and delusions of grandeur and persecution. Her depression had been treated with oral duloxetine 60mg twice daily, oral buspirone 10mg at night, and oral trazodone 150mg at night. She did have a urinary tract infection a week prior, but the psychotic symptoms persisted with antibiotic treatment. Miss X had also been on a monthly taper regimen of low-dose oral prednisone for RA (from 15mg to 5mg) for the past three months and had completed the regimen one week ago. On mental status examination, she was alert and oriented to time, place, and person. Her mood was irritable with lability. She demonstrated tangential speech along with persecutory and grandiose delusions. Attention and concentration was normal with intact immediate and remote memory and impaired recent memory. Abstract ability, judgment, and insight were impaired. Physical examination and vital signs were within normal limits. Laboratory investigations of complete blood count, urine analysis, urine drug screen, thyroid function panel, renal function panel, hepatic function panel, serum sodium, potassium, calcium, thiamine, vitamin B12, folate, and vitamin D did not show any significant abnormalities.

**Results:**

Miss X was admitted to the inpatient psychiatric unit with the diagnosis of medication-induced psychotic disorder, with onset after medication use as per The Diagnostic and Statistical Manual of Mental Disorders 5^th^ edition- Text Revision. Oral olanzapine 10mg at night and oral lithium 300mg twice daily was started along with her home medications of oral duloxetine 60 mg twice daily and oral trazodone 150mg at night. During hospital stay, oral olanzapine was gradually increased to 15mg in the night but had to be reduced back to 10mg in the night due to sedation. Miss X’s symptoms improved during hospital stay and she was discharged on the 13^th^ day of hospitalization with the same psychotropic medication regimen.

**Conclusions:**

Our case demonstrates the need for caution in prescribing steroids in older adults as it can precipitate neuropsychiatric symptoms even with a change in use or after withdrawal of steroids.

**Disclosure of Interest:**

None Declared

